# A novel genetic-artificial neural network based age estimation system

**DOI:** 10.1038/s41598-022-23242-5

**Published:** 2022-11-11

**Authors:** Oluwasegun Oladipo, Elijah Olusayo Omidiora, Victor Chukwudi Osamor

**Affiliations:** 1 Department of Computer and Information Sciences, Covenant University, Ota, Ogun State Nigeria; 2 Department of Computer Science and Engineering, Ladoke Akintola University of Technology (LAUTECH), Ogbomoso, Oyo State Nigeria

**Keywords:** Computational biology and bioinformatics, Genetics, Engineering, Mathematics and computing

## Abstract

Age estimation is the ability to predict the age of an individual based on facial clues. This could be put to practical use in underage voting detection, underage driving detection, and overage sportsmen detection. To date, no popular automatic age estimation system has been developed to target black faces. This study developed a novel age estimation system from the combination of a genetic algorithm and a back propagation (BP)-trained artificial neural network (ANN) and using the local binary pattern feature extraction technique (LBGANN) targeted at black faces. The system was trained with a predominantly black face database, and the result was compared against that of a standard ANN system (LBANN). The results showed that the developed system LBGANN outperformed the LBANN in terms of the correct classification rate.

## Introduction

Sufficient data describing human characters are displayed on the human face. Computer-based systems for predicting human traits, including gender, expression, feelings and intention, identity and race, have recently attracted much interest from researchers across the globe. Despite this attention, there are still many but to be discovered in the place of computer-based age estimation and prediction^1^. This is because aging differences indicate numerous inimitable capabilities, which makes age prediction a challenging task specifically for machines^2^.

Practically, face-based age estimation may be defined as the projected age of a person that relies upon or perceivable at the face image, typically the 2-D photo of the individual’s face^3^. In doing so, custom reference points referred to as fiducial points are manipulated to deduce the identification of someone and estimate the age. Studies such as craniofacial morphology, a branch of morphology that studies the shape of the face and skull, are limited, as they provide theoretical descriptions for various computing techniques to describe face images with age progression. People’s appearance can be substantially affected as age progresses due to adjustments to craniofacial morphology, face pores and skin pigmentation and face texture. Changes in texture may be deduced from pores and skin and muscle elasticity^4^.

Some craniofacial morphology capabilities and characteristics are precise to some age brackets and are altered through the aging process. Changes in skin pigmentation are predominant in growth noticeable from birth to adulthood, while skin texture modification can be seen at some stage in adulthood^5^.

Most age-related fraud is seen in the sub-Saharan African region. This could be a result of a lack of birth databases and documentation in rural areas in the region. Mothers still give birth outside the government hospital where a record of birth is lacking. With this, people could claim fraudulent age to obtain jobs meant for younger candidates, and sportsmen could claim younger age to claim sports engagement^1^. This could also have a life-threatening effect. As in the case of unworthy possession of driving licenses by the underaged candidate, which could lead to an accident^6^. The political effect is also seen in underage voting, a major problem in the electioneering process in the region.

Most literature in the area of face-based age estimation has used the FG-NET and MORPH II databases for training and testing^1^. These databases are scarcely populated with black faces. The other-race effect could make a system trained and tested using a face from a specific race underperform when put to use in a region with predominant people from another race^7^. This could be naturally explained by the memory representation of a child that has a greater capacity to recognize different people from the same race and have the feeling that people from another race all look alike. With this being said, there is a need to develop a system trained and tested with a predominantly black face database^8^.

A major limitation in this study is the data limitation. People between the ages of 40 and above 60 were not disposed to give out their face image alongside their age. They assume this could be used to compromise their official record, as many people documented younger age to become employed.

This was what motivated a shallow training paradigm for this study. Deep learning algorithms are known to be data-hungry and perform optimally with a large dataset. In this study, a local predominantly black face database was used in training and testing the developed system.

Some of the approaches that have been used for face-based age estimation include support vector machines, bioinspired features (BIFs), K-nearest neighbors, nonlinear aging pattern subspace and neural network approaches^9^. However, optimization techniques can be further integrated into the classification module of age estimation systems to improve its overall performance.

Back propagation (BP) is an algorithm commonly used in training multilayer networks, despite its drawback of a high tendency to converge at local minima. BP is usually combined with gradient descent as an optimization technique. BP iterate through a two stages. Namely, the propagation and weighing apprise. The network’s input is propagated forward passing through all layers until it reaches the output layer^9,10^.

A loss function is adopted to compare the network’s output with the anticipated output. Additionally, inaccuracy values are computed for neurons in the output layers. These inaccuracy values are propagated backward so that each neuron will have an ascribed error value to indicate its part to the original output. BP during the weighting update phase uses the computed error values to determine the gradient of the loss function with respect to the network^11^. The gradient serves as input to the optimization technique and is in turn used to update the network’s weight to minimize the loss function.

BP has proven to be proficient when deployed to classification problems, which motivated^9^, who developed a face age estimation system using principal component analysis (PCA) and BPNN. The study displayed an improved age estimation accuracy but still suffered pitfalls from scaling problems. Multilayer perceptrons trained using standard BP perform well in cases of noncomplex training problems. However, as the difficulty in the problem landscape increases, the performance of BP drastically decreases. This is due to the existence of a global minimal sandwiched around the local minimal in such problem space. The gradient descent optimization technique could be trapped at local minima when close global minimal is concealed amid local minima, BP has the tendency to end up bouncing among local minima with no significant overall progress, which could result in slow training.

Researchers are now tailored to improving BP to ensure its convergence at the global minimum. Several adjustments to conventional BP have been developed, such as modified backpropagation^12^ and optical backpropagation using squared error^13^. Additionally, rigorous studies have been performed, resulting in new training modalities for multilayer network training. The studies in^9,14^ achieved an improved number of epochs, convergence time, learning rate and better weight adjustment. Modified optical back propagation (using cubic error adjustment) developed by^15^ has a better advantage over the existing systems in the literature).

Furthermore, in this domain, this study aims to develop a face-based age estimation system using a genetic algorithm (GA)-back propagation neural network (BPNN) for better age estimation and a local binary pattern for feature extraction to encode texture and appearance information. The combination is motivated by the fact that GA has the potential to traverse the entire search space while remaining time-efficient. Hence, it would offset the problem of BP being trapped in local minima.

Feature extraction involves the transformation of image features into a whole new feature content. During feature extraction, features used for selection and classification tasks are produced. Feature reduction and selection help reduce redundant features and present only discriminative features for the classification task.

**Figure 1:**
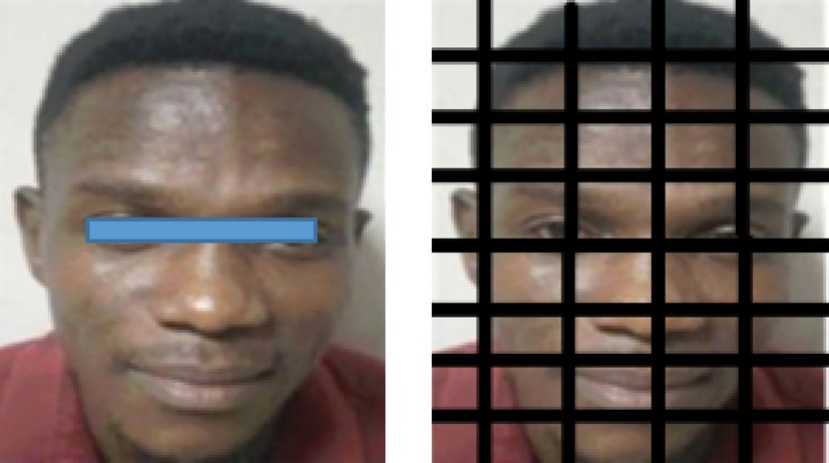
Face image divided into different sections.

LBP is a relatively new technique for feature extraction. It was introduced by Ojala ^16^ that LBP makes it possible to depict both texture and shape information of an image. This was made possible by dividing the image into numerous smaller sections and extracting features from these sections. This is shown in Fig. 1.

LBP is rich in shape and texture features. These capabilities of LBP motivate its choice as the feature extraction technique used in this study. Hence, this study aims to develop a novel age estimation system using the combination of a genetic algorithm (GA) and back propagation artificial neural network (ANN)-based methods and LBP for feature extraction.

The following are the specific objectives of the study.

- Develop an age estimation system by hybridizing a genetic algorithm-artificial neural network (GA-ANN) and LBP for feature extraction
- Simulate the developed system in (i) using object-oriented MATLAB programming language.
- Access the performance of the developed model based on the correct classification rate (CCR), viz-a-viz a standard back propagation artificial neural network-based age estimation system.

## Related works

Age estimation can be summarized as biometric-based operations involved in predicting human age or age range in this case based on facial characteristics and inherent facial features^7^. This, unlike other aspects of face recognition application, has not been popularly researched, despite its wide and promising application. The application of age estimation could be deployed into age-based access control, age-specific human computer interaction to specify preference for different age groups, source of data to infer actions Internet of Things (IoT) configurations, social media minor control systems (internet safety for minor), law enforcement and surveillance (such as age limitation and control in traffic), alcohol dispensing system control and other host of things^1^.

**Figure 2:**
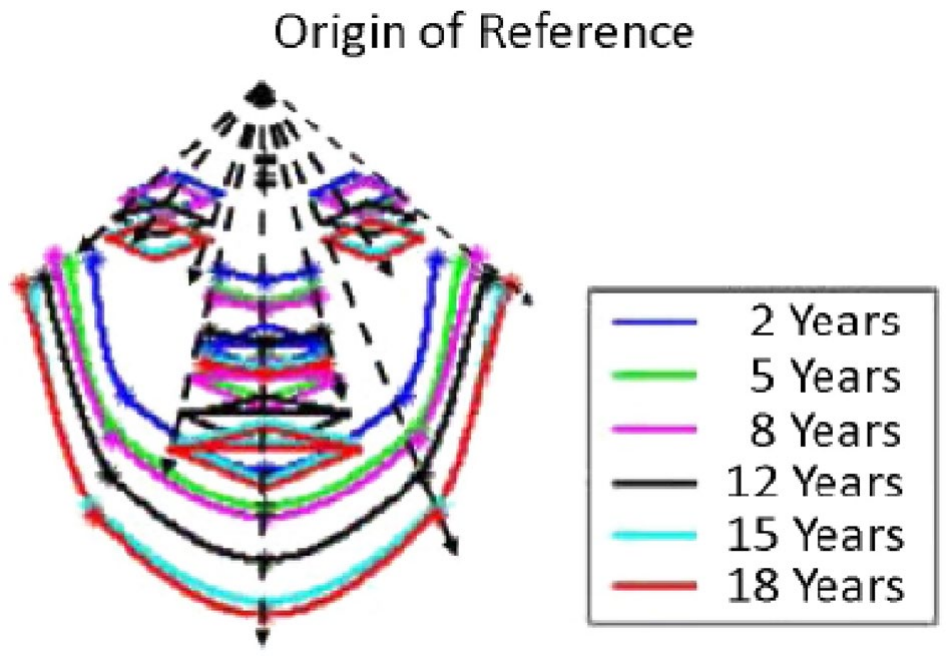
Cranio-facial changes during the first phase of aging.

**Figure 3:**
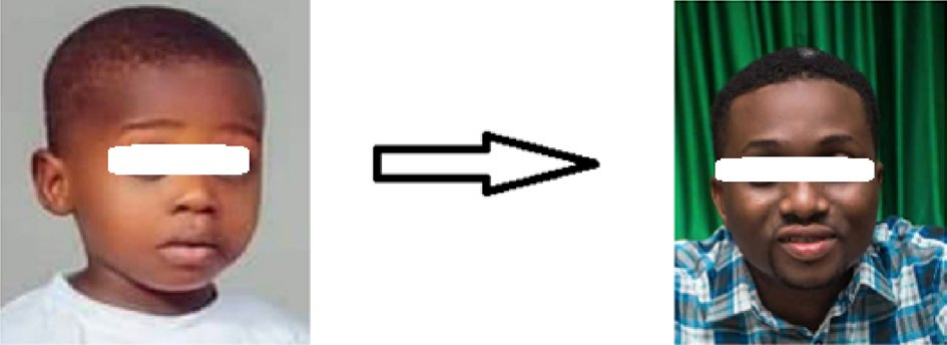
Skin and facial hair during the first stage of aging.

In estimating age using computers, it is imperative to study the process of natural aging. According^7^, there are two recognized stages of face-expressed aging. The first phase is experienced during early years. This covers from birth to adulthood. During this stage, cranofacial changes accounted for most of the changes, as shown in Fig. 2. These changes include a more pronounced chin, cheeks brooding over a larger area, forehead falls back to reduce the free space on the surface of the skull, and face characteristics amplify and cover the interstices. In addition to these changes, there were also minor changes seen in the skin color and facial hair. The hair becomes heavy and denser and changes color, as shown in Fig. 3.

The other phase is observable during adulthood. This spans from the time growth stops (only little growth could be noticed) until old age. Most changes in this phase are seen in the skin texture; the skin becomes thinner, darker, less elastic and more rugged. In addition, wrinkles start to show, under chin become prominent, sagging cheeks and lowered bags beneath the eye begin to manifest. Although craniofacial changes are pronounced during the first stage, little is noticeable during this stage^17^. The U-shaped face gradually grows into a trapezoidal form, while the upside down triangular face grows rectangular^1^.

### Age estimation pipeline

The pipeline according to^1^ commences with the image preprocessing stage. This includes face detection, face alignment, face image cropping, and grayscale conversion. The face age model represents the face image using techniques such as the Anthropometric Model, Active Appearance Model, AGing pattErn Subspace and Age Manifold Model. The age model classifier and algorithm are similar to the face age model used but can also be seen as a special pattern recognition problem that categorizes age estimation into classification problems or regression problems. It is a classification problem when class labels are used (e.g., adult, minor, senior adults, above 60 years) and a regression problem when age is resolved to be a number^7^. Some classification methods used in age estimation include the nearest neighbor approach, multilayer perceptron and self‐organizing map (SOM). When age estimation is treated as a regression problem, the aging function is linear, quadratic, and cubic and can be used to estimate age^18,19^. These all work together to estimate the age or age group of a probe face image.

### Related works

Lanitis^20,21^ create a two-step model that is classification-centered. The first step involved identifying the gender and race of each face, after which the age of each race was calculated. In^22^, an ordinal classification model was used to predict age. According to their relational order, the facial features are divided into groups using positioned decision boundaries^23^. By combining organized references from the placed decision boundaries, the ages are inferred.

In a similar manner^7,24^, used a hierarchical model for age prediction and investigated how features taken from a 3-D face image were affected by aging^25^ overcame a common age estimation research limitation of sparse and unbalanced datasets by developing a system for crowd density and age prediction. In the presence of imbalances in the face image dataset, the developed system could make use of the cumulative attribute approach to learning a regression model.

Demontis^26^ provided a sparse regression method that was honed using the Face Recognition Grand Challenge database in his study. FG-NET database images were used to test the final model^27^ used manifold learning and deep learning architecture to estimate a person's age. The convolutional network was used to extract features, and face aging features were obtained at more than just the top layer of the system developed^28^. In order to improve the age estimation system's accuracy^29^, also investigated the use of a convolutional neural network (CNN).

Oladele^11^ created an artificial neural network with back propagation that estimates age using data from the FG-NET database. In this study, face images were divided into eight (8) age categories, and appearance and texture information were represented using principal component analysis^30^ examined the effectiveness of age estimation systems created with a self-organizing feature map and a back propagation artificial neural network, both of which are unsupervised learning paradigm neural networks. The performance of the two aforementioned systems was statistically compared in the study using principal component analysis for feature extraction. The study's conclusion demonstrated that the self-organizing feature map outperformed the artificial neural network that had been trained using back propagation.

Chen^31^ demonstrated how the use of a big database and deep learning algorithms can enhance the performance of an age estimation system. A ranking CNN model that combines a fundamental CNN with a series architecture was suggested by the study. The final output of the CNNs is an aggregation created from the binary output of the individual CNNs. The CNN content is trained with ordered age labels. It was determined that ranking-CNN could outperform traditional CNN models.

Li^32^—used a cumulative hidden layer strategy to address the problem of imbalances in image datasets that are present in large databases. In the model, pairwise relative signals are used in the supervision of the comparative ranking layer to support learned age features using faces from similar age groups. The implemented ranking layer encourages aging feature learning and enhances the age estimation of the entire model.

By using face images and speech recognition for age estimation^33^, proposed a multimodal approach to age and gender estimation. Two joint deep neural networks were trained using depth information derived from the face image and both appearances. Along with that, the mel frequency cepstral coefficient that was extracted from speech samples is used. The joint deep neural network was fine-tuned using a novel cost function to increase accuracy and decrease overfitting overhead.

Ji^34^ noted that fluctuation might be experienced when using face images in a video frame to train a traditional convolutional neural network for age estimation. In addition to the convolutional network, an attention mechanism was used. As an encoded feature for age estimation, this model represents an attention chunk that contains an aggregated feature space. To improve the age estimation of the images in the frame, the stabilization of the frames is accomplished using a novel loss function.

For the classification of age, gender, and race^35^, used a particular autoencoder. Duan et al.^35^ created a hybrid system in their study by fusing a CNN with an extreme learning machine (ELM). The ELM was used to classify subjects into different age ranges, and the CNN was used to extract age-related features from the used face images. The audience database and the well-known MORPH II database were used in the study's training and testing of the created system^36^. Used a cascaded structured model that took advantage of the relationships between secondary demographic evidence like race and gender. Learning all the frameworks embedded in the parent network and child networks is made easier with the help of the secondary information. Compared to the previous age estimation system, this increased the accuracy of age prediction.

Angulu et al.^37^ combined local discriminant analysis (LDA), LBP, and Gabor filters to represent the face image's appearance, shape, and texture in order to estimate age. The study also used a combination of support vector machines (SVMs) and artificial neural networks (ANNs) to classify the face's age into different age ranges. An age label was computed from the combination of global and section-built matchers and assigned after the ensemble was directed by the majority voting scheme.

Nam^38^—took note of pictures taken with mobile phones in real-world situations and settings that were not under control. Due to the resolution of the phone camera, such images are of lower quality. To create a high-resolution reconstruction, the images are fed into a conditional generative adversarial network (GAN) model. The study^39^ used SVM as a classifier with LBP and FSM feature extraction techniques to represent face images and CNN for age estimation with PAL and MORPH face image databases for training and testing. The age estimation system's performance was enhanced by the two-way feature selection.

Observing the developments in the field of automatic age prediction: Although deep learning techniques appear promising, it is well known that they struggle when trained with small datasets. In the Sub-Saharan African region, gathering face image data is time-consuming and hectic, sometimes even impossible with some age groups. This is the case with subjects in the age group of "over 40". They resented enrolling their faces. As some people claim an age that is lower than their actual age in order to secure jobs that are available for lower ages, they are worried that their true age will be discovered and will conflict with their official record at work.

The study uses a genetic algorithm back propagation trained artificial neural network-based classifier for age group classification to get around the problem of not having enough black faces. Additionally, it made use of a subset of FG-NET database data (selected 500 images).

## Methodology

The study presents the development of a genetic algorithm and artificial neural network (GA-ANN)-based age estimation system. The motivation for the choice of the algorithm was the nature and the quantity of data. Face aging presents many unique attributes that vary from person to person. The ability of ANNs to learn without serious knowledge of the features to be learned and the flexibility offered by the weighing adjustment make them a suitable choice for other machine learning tools^40^. The quantity of available data, which is low and inadequate to support a deep learning paradigm, also makes backpropagation a good choice. Part of the novelty of this work is the modality of combining the GA to optimize the ANN for face-based age estimation, the method of encoding the chromosome, and implementing the crossover operator. In this work, GA is employed to intelligently direct search for an optimal solution by presenting a GA-ANN object with the highest fitness.

**Figure 4:**
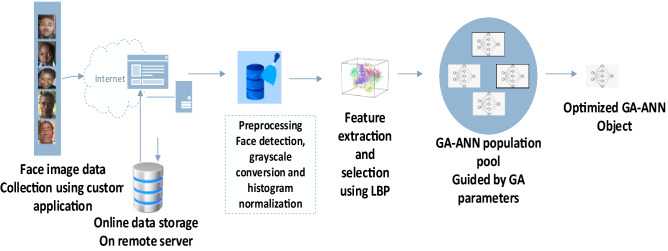
Developed system framework.

The developed age estimation system was implemented in a MATLAB object-oriented programming environment. MATLAB is a scientific programming language that provides strong mathematical and numerical support for the implementation of advanced algorithms. Figure 4 shows the developed system architecture.

### Image acquisition

**Figure 5:**
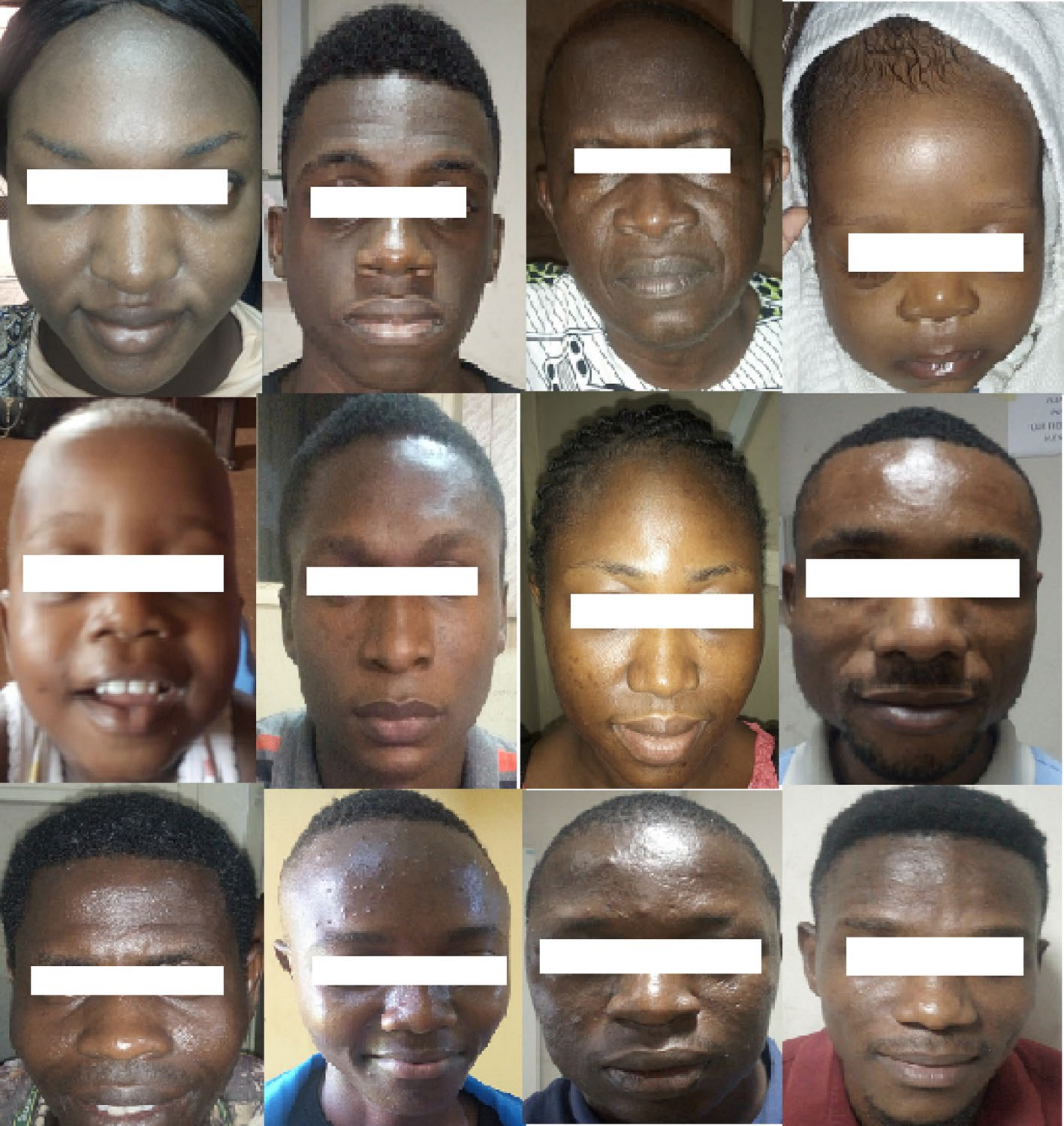
Sample images from developed face database.

The image database developed in this study was populated with 1355 images. A total of 855 of the images were sourced locally from more than 800 subjects, and 500 images were selected from the FG-NET database to complement the locally sourced images. This was done to ensure ample images are available across various age bracket to train and test the developed AES. Figure 5 shows sample images from the image database. The age groups and the numbers of images per age group in the database are shown in Table 1.

**Table 1:** Developed database age distribution.

Age Group	Local images	FG-NET Images	Total
0–5	109	91	200
6–10	103	86	189
11–20	250	113	363
21–30	118	82	200
31–40	90	68	158
41–50	70	39	109
51–60	57	14	71
Above 60	58	7	65

Face images were captured under various illumination conditions. Slight head rotation and cosmetics were allowed to depict real-life situations. The database for this research comprised images of various sizes, background information, lightening, and complex head poses, which are usual in an uncontrolled environment.

**Figure 6:**
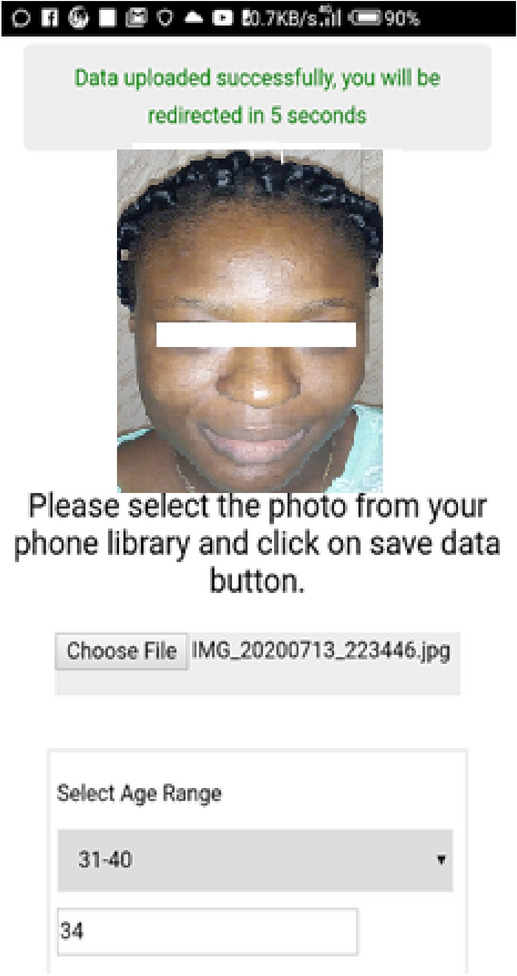
Face image acquisition mobile application.

It should be noted that a custom mobile application was developed for data collection to ensure that distance to candidates who wished to enroll was not a problem. The mobile application was developed using JavaScript, HTML5 and PHP programming languages. The developed application was compiled using CORDOVA to make it available on various mobile platforms. The face image acquisition interface is shown in Fig. 6.

It is good to note that informed consent was obtained from all subjects and, in cases where subjects are under 16, from a parent and/or legal guardian. This is in conformance with the policies guiding human subject-based research conducted at Covenant University by the Covenant Health Research Ethics Committee (CHREC). All the simulation procedures carried out in this study were approved by CHREC. The study was carried out in accordance with the guidelines and regulations for conducting human-subject related research as laid down by CHREC. It is also good to note that informed consent to publish was obtained from all participants**.**

### Image pre-processing

The unwanted data and background information are removed during the preprocessing and normalization stages. In this stage, the acquired images were converted to grayscale format, and then the face area useful for recognition was automatically detected and cropped using the viola jones algorithm. The outcome of this was subjected to histogram equalization. Histogram equalization was employed to increase the image contrast and reduce the effect of uneven illumination since the image data were collected in an uncontrolled environment.

**Figure 7:**
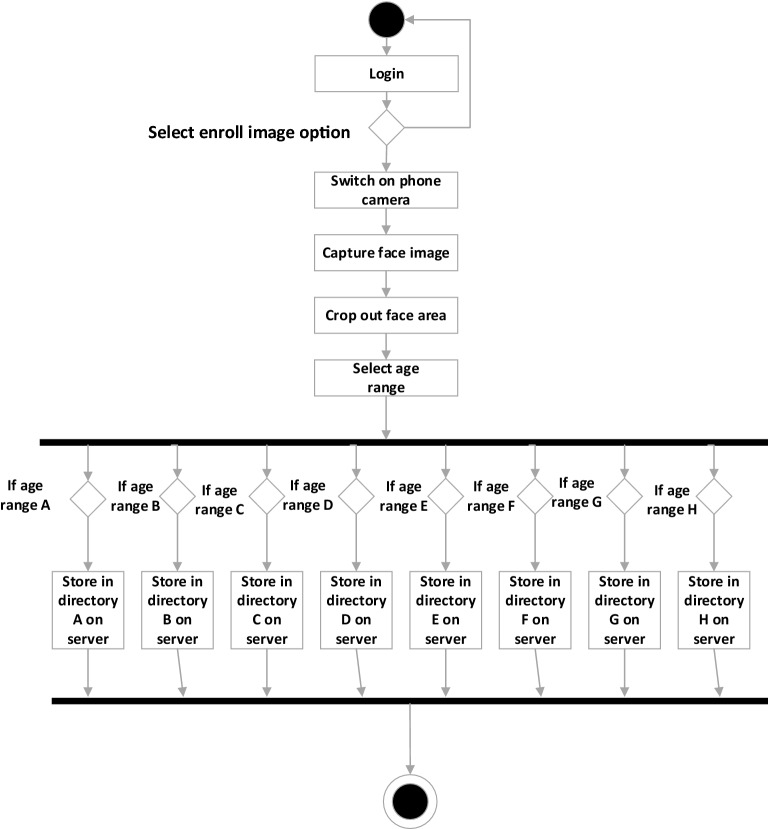
Activity diagram for the image acquisition module.

A UML activity diagram to model the face image acquisition module is shown in Fig. 7.

### Feature extraction

In this study, the LBP feature extraction technique was deployed to extract age-related information for training and classification of face images. The LBP features generated were subjected to feature selection using principal component analysis (PCA).

A 3 × 3 pixel image with a center pixel (x_c_,y_c_) intensity value g_c_. the local texture is computed as T = t(g_0_…,g_7_), where g_i_(i = 0…,7), which corresponds to the gray values of the 8 surrounding pixels. These surrounding pixels are thresholded with the center value g_c_ being t(s(g_0_ − g_c_)…, s(g_7_ − g_c_)) and the function s(x) being defined in Eq. (1). Then, the LBP pattern at a particular pixel was obtained using Eq. (2)^41^.

1 $$S\left( x \right) = \left\{ {\begin{array}{*{20}c} {1,} & {x > 0} \\ {0,} & {x \le 0} \\ \end{array} } \right.$$

2 $$LBP \left( {{\text{Xc}},{\text{ Yc}}} \right) = \mathop \sum \limits_{i = 0}^{7} 8\left( {gi - gc} \right)2^{{\text{I}}}$$

An image space is a space with the number of pixels as dimensions. By converting the image to an image vector using column concatenation, the image can be treated as a point in the image space. When all the training set images are converted into vectors, they categorize similar and fixed features such as eyes, nose and mouth into a specific location. The eigenface method starts with this correlation and tries to find lower dimension space for the face images by focusing on the variation between face images^41^.

The feature vectors (I) from LBP serve as the training set for the PCA method. When M is the total number of images in the training set. The deviation of each image from the mean image is calculated from Eqs. (3) and (4).

3 $$\uppsi = \frac{1}{M}\mathop \sum \limits_{n = 1}^{M} {\text{In}}$$

4 $$\varphi_{{\text{n}}} = {\text{I}}_{{\text{n}}} {-}\psi$$

The variation among the eigenvectors of the covariance matrix is calculated using Eq. (5). The space where all these eigenvectors reside is called eigenface space or eigenspace. All the training set images are projected into the eigenface space using Eq. (6).

5 $$C = \frac{1}{M} \mathop \sum \limits_{n = 1}^{M} \varphi n\varphi Tn = AA^{T}$$

6 $$\omega_{k} = u_{k } .\varphi = u_{k} .\left( {I - \psi } \right)$$

The weight matrix Ω = [ω1,ω2…,ω0 M]T was the representation of a training image in the eigenface space. In this study, LBP is implemented in the steps highlighted in Algorithm 1.
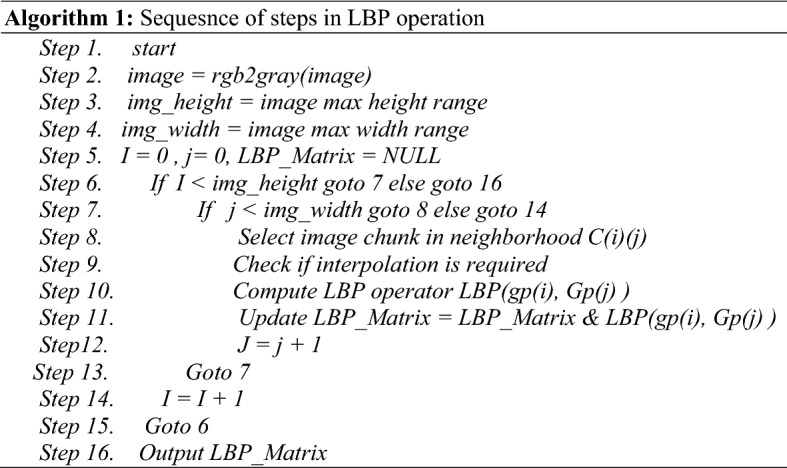


### The hybridized Genetic Algorithm-Artificial Neural Network (GA-ANN) classifier

**Figure 8:**
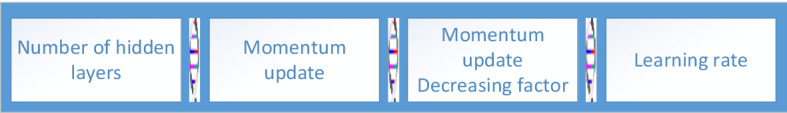
Sample GA-ANN Chromosome.

**Figure 9:**
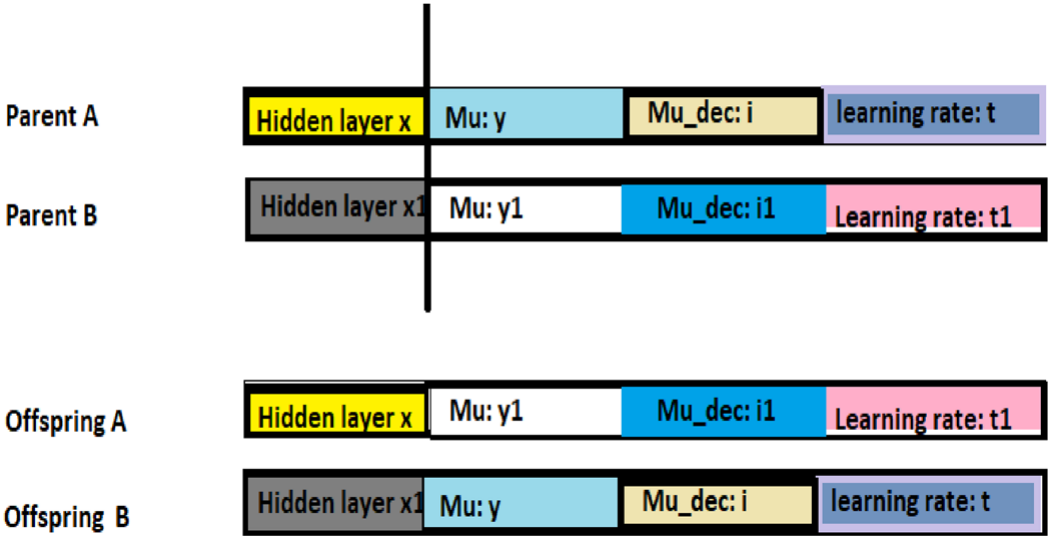
Single-point crossover implemented in the GA-ANN Classifier.

The GA-ANN is a combination of a genetic algorithm (GA) and an artificial neural network (ANN). The combination is motivated by the ability of GA to intelligently direct search to discover the optimal solution by preferring chromosomes with high fitness. The combination architecture employs encoding of ANN parameters as GA chromosomes. The ANN parameters employed for this are the numbers of hidden layers, the network momentum update (MU) and the decreasing factor of the MU, i.e., MU_dec as seen in Fig. 8. Single point crossover was adopted. As such, a random point is selected on the parent chromosome, and genes to its right are interchanged to generate new offspring (see Fig. 9).

**Figure 10:**
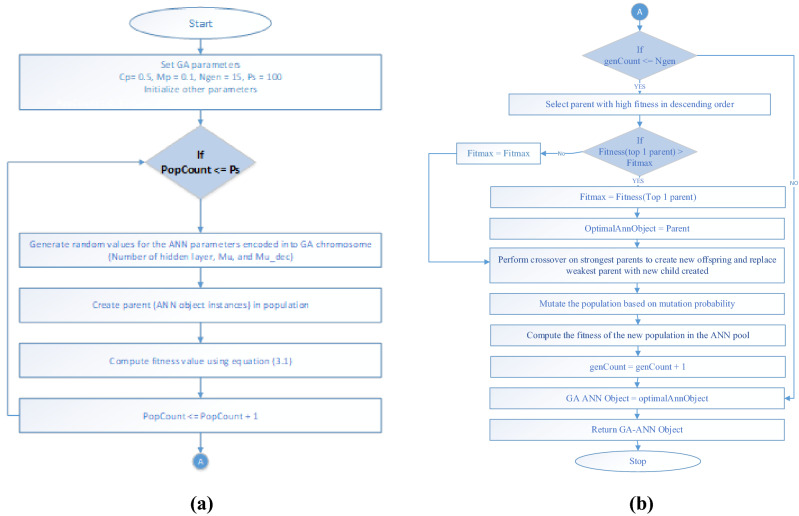
(**a**) First stage flowchart of the GA-ANN training phase. (**b**) Second stage flowchart of the GA-ANN training phase.

GA parameters such as population size (Ps) was 100 and number of generations (Ngen) was set to 25, mutation probability (Mp) was set to 0.1 and crossover probability (Cp) was set to 0.5.

The system stopping criterion is the total number of iterations as specified by the number of generations (Ngen). Fitness was computed using Eq. (7), the inverse of the ANN mean of squared errors (MSE),

7 $${\text{Fitness}} = \frac{1}{MSE}$$

The GA-ANN ANN's object compartment was trained using 80% of the database images, as specified by the function "net.divideParam.testRatio." This was done to ensure that the various variations in age estimation were well captured. Algorithm 2 depicts the GA-ANN training phase algorithm, and Fig. 10a,b depict the flowchart.

The flow chart depicts the various generations and manipulations that take place in the GA-ANN module of the developed AES. There are 100 ANN objects in the initial population. To compute fitness, the ANN objects are subjected to Eq. (7). The lower the MSE, the more fit the neural network object. The neural network objects that result are arranged in descending order of fitness. On the fit objects, a recombination operator is applied, and a mutation operator is applied based on random mutation tendency. A new generation is formed and chosen to be the next generation's population. The number of generations is used as the stopping criterion, and the neural network object with the highest fitness is used for classification. The GA-ANN model configuration is shown in Table 2.

**Table 2:** GA-ANN model configuration.

GA-ANN model parameters	Description	Values
trainFcn	Training function	Trainlm
hidLayer	Numbers of hidden layer	None zero auto tune by GA
performFcn	Performance function measure	MSE
trainParam.goal	Stopping criteria	1e-3
divideFcn	Data divide function	Divideblock (to avoid overlapping training and testing data)
divideParam.trainRatio	Dataset training ration	80/100
divideParam.testRatio	Dataset test ration	20/100

It should be noted that each neural network is encoded as a chromosome, with the various genes being the number of hidden layers, learning rate, and momentum updates. The values of the aforementioned gene are computed using a genetic algorithm method that tunes the ANN object. It should also be noted that having to automatically adjust the ANN's control parameters Mu dec and learning rate aids the GA-ANN in dealing with imbalance data, which is caused by the scarcity of face images in some age groups.

The GA-ANN computes fitness based on the MSE of the ANN object and re-tunes the neural network parameters until it finds the network with the highest fitness of the ANN pool that was iterated through generations based on the value of "Ngen," which is set to 25 due to the available computational resources used during the simulation.
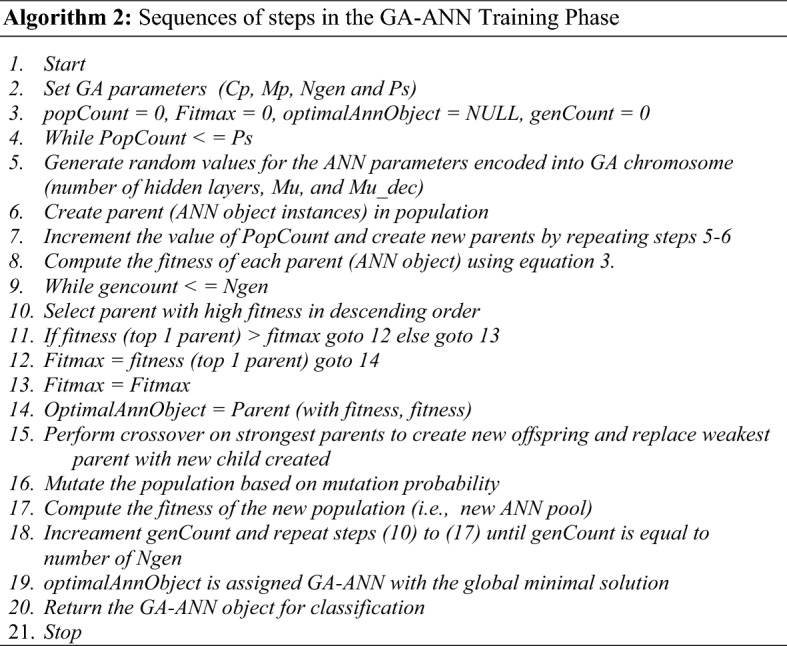


### Testing and performance evaluation

**Figure 11:**
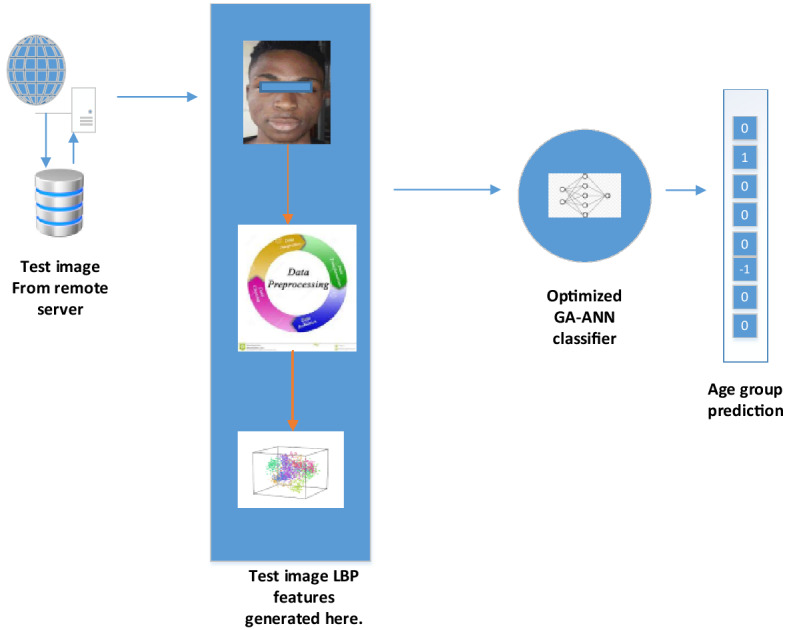
GA-ANN AES testing framework.

**Figure 12:**
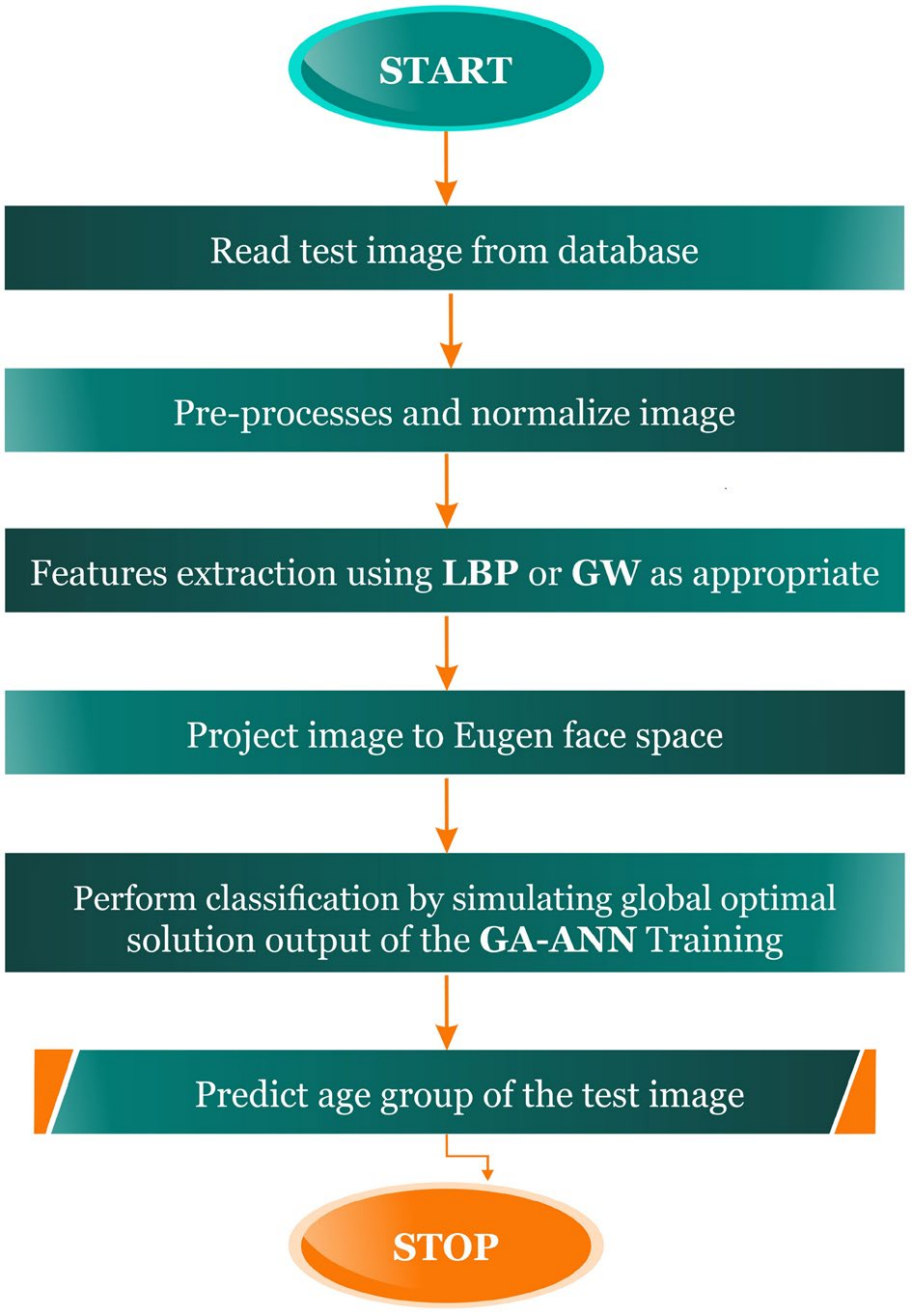
The developed GA-ANN-based age estimation system's process pipeline.

170 images were separated and stored on a remote server for testing that had not previously been used in training the developed GA-ANN age estimation system. The images are preprocessed and feature extraction is performed. The mean of the LBP feature is subtracted and then projected to the eigenface space. The extracted features are fed into the GA-ANN system for classification, and Fig. 11 illustrates the testing framework. The pipeline of the developed system can be summarized in the flow chat in Fig. 12.

The performance evaluation of the developed age estimation system (AES) was performed by comparing the correct classification rate (CCR). This is because of its ease of clarification in discriminant analysis, the correct classification rate is a preferred valuation measure for classification problems. It is also worth noting that the correct classification rate does not necessitate the use of domain-specific information in its computation. As a result, this metric can be used to assess the classification accuracy of various models. As being such, it would aid in determining the classification accuracy of the developed age estimation system in this study. This would be used to compare the accuracy of the developed GA-ANN-based system to the accuracy of a standard ANN-based age estimation system. The CCR is computed using Eq. (8)

8 $${\text{CCR}} = \frac{1}{n}\mathop \sum \limits_{i = 1}^{n} \delta \left( {{\text{a}},{\text{ A}}} \right)$$

‘*δ*’ is a pointer variable such that *δ*$\left( {a, A} \right)$ is computed as 1 if a = A and zero when a <  > A.

The computed CCRs for the developed GA-ANN-based AES (LBGANN) and the standard ANN-based AES (LBANN) are subjected to one-way ANOVA to determine whether the improvement of the developed system is statistically significant. Minitab software was used for this. To elaborate on the performance evaluation, the GA parameter "Ps" was tuned by increasing the population size by 100 increments over time. This is done to more accurately reveal the neural network object, which is a result of GA operator manipulations. Also, the CCR of the developed LBGANN was compared to the AES developed using the Wavelet Decomposition (WD) feature extraction technique to further examine the performance of LBP feature extraction used in the LBGANN system. The output was tested using one-way ANOVA to see if it was statistically significant.

## Results and discussion

The GA-ANN-based AES with the LBP feature extraction module is represented as LBGANN in this section of the study, while the standard ANN-based AES with LBP feature extraction is referred to as the LBANN system.

### Comparative analysis of LBGANN and LBANN-based AES

The results of the LBGANN and LBANN system simulations carried out for different age groups are shown in Table 3. The LBGANN and LBANN correctly classified 156 and 144 images, respectively, of the 170 test images and showed CCRs of 91.76% and 84.71%, respectively. This shows that the LBGANN outperforms the LBANN in terms of CCR.

**Table 3:** Correct classification rate of LBGANN and LBANN.

Age range	Test images	LBGANN		LBANN	
		Correctly classified images	CCR (%)	Correctly classified images	CCR (%)
0–5	20	18	90.00	17	85.00
6–10	20	18	90.00	17	85.00
11–20	30	30	100.00	28	93.33
21–30	30	27	90.00	24	80.00
31–40	20	19	95.00	17	85.00
41–50	20	18	90.00	16	80.00
51–60	15	13	86.67	12	80.00
Above 61	15	13	86.67	13	86.67
Total	170	156	91.76	144	84.71

**Figure 13:**
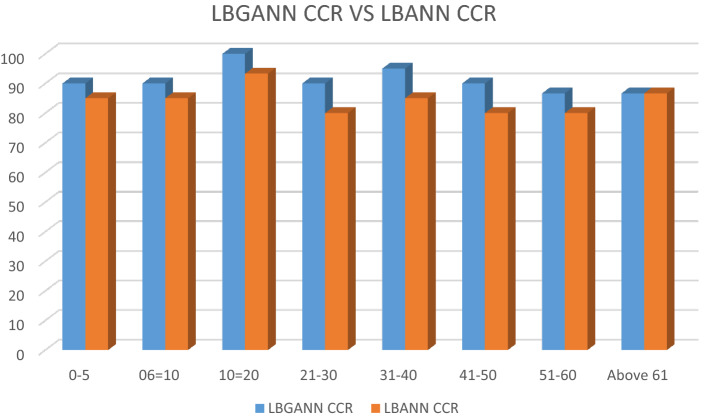
LBPGANN CCR and LBANN CCR.

The LBGANN and LBANN systems recorded the best classification rate in the images of the (11–20) age group. This is due to the high percentage of face images in the 11–20 category of the training database. Figure 13 shows a 3D column chat of the CCR of LBGANN and LBANN. Additionally, according to^7^ and^17^, the age group mainly belongs to the first phase of face-expressed aging, and this phase reveals many craniofacial changes that are held in generating rich LBP features. The graph of the CCR for LBGANN and LBANN is represented in Fig. 12. The LBPGANN has its minimum CCR of 86.67% at age ranges 51–60 and above 60.

Table 3 also reveals that there was a decrease in CCR within the age groups (31–50) and those above 60 due to wrinkle and skin lethargy, which considerably altered their textural features. Wrinkles on the human face increase as one grows, particularly those within the age bracket of [30 to 80]^11,17^. As a result, those in the age group ‘above 60’ had significantly more wrinkled faces, which inhibited the discriminant ability of the textural features encoded using the LBP technique. The age range (31–40) had robust textural features with fewer wrinkles, accounting for the high CCR value of 95%.

Age groups 0–5 and 6–10 also showed a good CCR of 90%. This is because the age group possesses rich shape features that are a product of the craniofacial morphological changes evident in the first stage of face-based aging expression^42^. LBP is rich in depicting such shape features, which account for the high CCR for this age group. The LBGANN outperforming the LBANN system indicates that the genetic algorithm has the ability to direct a backpropagation ANN object to solution with minima errors for better performance.

### Statistical analysis

H0 = There is no significant improvement in the CCR of LBGANN and LBANN.

H1 = There is significant improvement in the CCR of LBGANN over LBANN.

Significance level = 0.05.

The result of the statistical test is shown below.
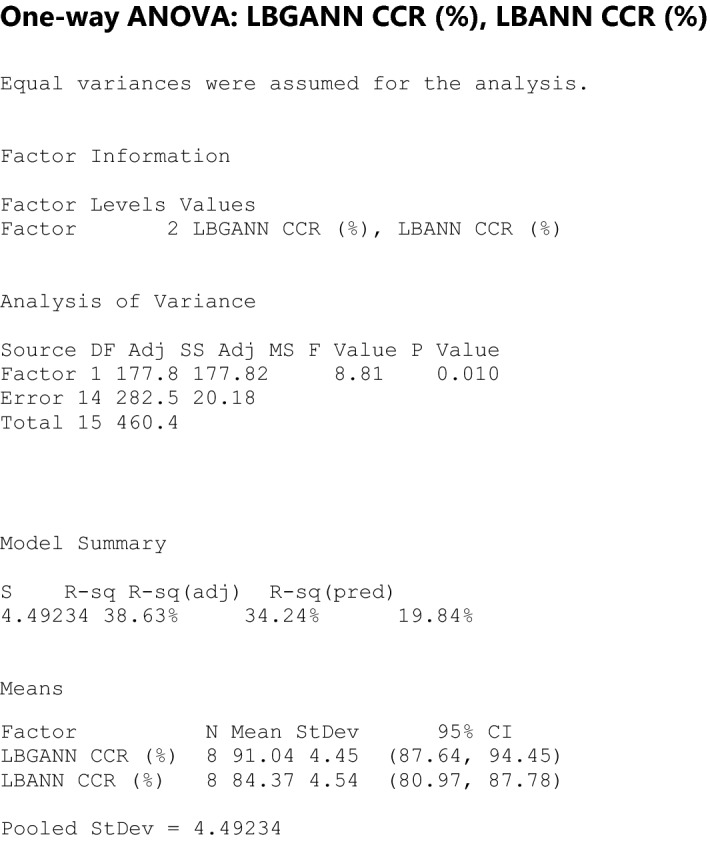


The *P* value (0.010) in the result of the analysis was less than 0.05. Hence, the alternative hypothesis is accepted, and the null hypothesis is rejected. This implies that the improvement in the correct classification rate of LBGANN over LBANN is statistically significant.

### Comparative analysis of LBP and wavelet decomposition in the developed AES

The Wavelet Decomposition (WD) feature extraction technique was deployed with the developed GA-ANN AES. The CCR at different age groups was computed and compared with the CCR shown with the developed LBGANN-based AES. Table 4 shows the result of the WD system side by side the developed LBGANN-based AES.

**Table 4:** Correct classification rate for LBGANN and WD GA-ANN AES.

Age range	LBGANN (CCR (%))	WD GA-ANN AES (CCR (%))
0–5	90.00	80.00
6–10	90.00	85.00
11–20	100.00	90.00
21–30	90.00	86.67
31–40	95.00	85.00
41–50	90.00	85.00
51–60	86.67	80.00
Above 61	86.67	80.00
Total	91.76	84.71

**Figure 14:**
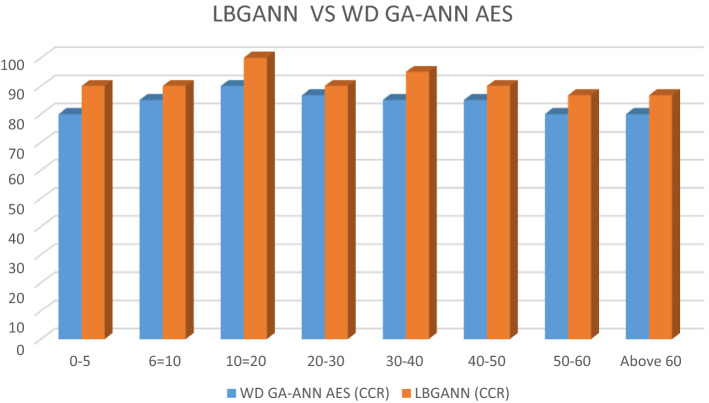
CCR for LBGANN and WD GA-ANN AES.

The 3D column in Fig. 14 shows the various CCR at different age groups for the LBGANN and WD GA-ANN age estimation systems. The developed LBGANN AES performed better than its WD counterpart as it had a total of 91.76% CCR. When the WD-based AES showed a CCR of 84.71%.

### Statistical analysis

When the computed data in Table 4 is subjected to one-way ANOVA, the result is as shown below.
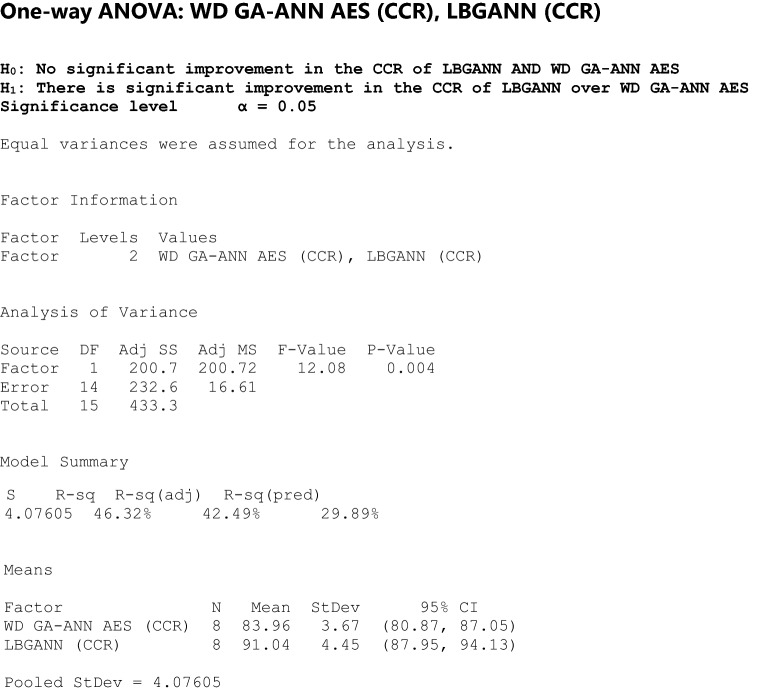


The statistical analysis revealed that the p-value is less than 0.05, implying that the null hypothesis should be rejected and the alternative hypothesis accepted. This clearly shows that the improvement in CCR demonstrated by the developed LBGANN AES is statistically significant when compared to the CCR of the WD GA-ANN AES.

### Comparison with other related works

The developed system was fine-tuned by adjusting the GA-ANN module's initial population size (Ps) parameter. The Ps parameter was varied between 100 and 500. This was done to vary the neural network object input biases and intelligently guide the pool of neural networks to produce GA-ANN object with optimal accuracy. Table 5 shows the CCR at various Ps values, with the number of generations set to 25, and the mutation probability and cross-over tendency set to 0.1 and 0.5, respectively.

**Table 5:** Correct classification rate at various Ps values.

Ps	LBGANN (CCR (%))
100	91.76
200	92.94
300	92.35
400	93.52
500	94.70

The developed AES, LBGANN, shows a better CCR of 94.70% than previous works in literature, such as that of Hasan and Mahdi^39^, with a correct classification rate of 93.81 on a collection of local data complemented with FG-NET data. The developed LBGANN AES also showed a better classification accuracy compared to Oladele^11^ when tested with a few sets of black faces, showing a CCR of 82.2% after training a PCA backpropagation artificial neural network with FG-NET data. Oladele^30^ also featured black faces in testing self-organizing feature map-based AES and showed a recognition accuracy of 92.2%, which is lower compared to the developed LBGANN AES in this study.

## Conclusion and recommendation

This study presented the development of an automatic age estimation system using face images. The LBGANN AES used a novel classifier that is a hybrid^43^ of the genetic algorithm and artificial neural network. The local binary pattern feature extraction technique was used, and a new dataset of predominantly black faces was developed for the study. The database contains 855 black faces and is complemented with 500 faces from the FG-NET database to make of a training database of 1355 images. The results showed that the developed LBGANN performs better than LBANN in terms of the correct classification rate and established that the genetic algorithm has the ability to intelligently search through a population of ANN objects to produce a fittest solution with better performance.

## Data Availability

The datasets generated and/or analysed during this study are not publicly available due to the private nature and the sensitivity of subjects' ground truth age of subject candidates. Despite having given their consent for the purpose of research, candidates still empathized the need to securely keep the face images and not make them available in the public domain, which could compromise their respective official records. In the case of reasonable request, the dataset could be made available by the corresponding author.
